# Appraisal of the Fairness Moral Foundation Predicts the Language Use Involving Moral Issues on Twitter Among Japanese

**DOI:** 10.3389/fpsyg.2021.599024

**Published:** 2021-04-30

**Authors:** Akiko Matsuo, Baofa Du, Kazutoshi Sasahara

**Affiliations:** ^1^Department of Psychology, Tokai Gakuen University, Nagoya, Japan; ^2^Department of Complex Systems Science, Graduate School of Informatics, Nagoya University, Nagoya, Japan; ^3^Department of Innovation Science, School of Environment and Society, Tokyo Institute of Technology, Tokyo, Japan

**Keywords:** moral judgment, SNS, moral foundations dictionary, culture, Japan, Twitter, language, morality

## Abstract

Moral appraisals are found to be associated with a person’s individual differences (e.g., political ideology), and the effects of individual differences on language use have been studied within the framework of the Moral Foundations Theory (MFT). However, the relationship between one’s moral concern and the use of language involving morality on social media is not self-evident. The present exploratory study investigated that relationship using the MFT. Participants’ tweets and self-reported responses to the questionnaire were collected to measure the degree of their appraisals according to the five foundations of the MFT. The Japanese version of the Moral Foundations Dictionary (J-MFD) was used to quantify the number of words in tweets relevant to the MFT’s five moral foundations. The results showed that endorsement of the Fairness and Authority foundations predicted the word frequency in the J-MFD across all five foundations. The findings suggest that the trade-off relationship between the Fairness and Authority foundations plays a key role in online language communication. The implications and future directions to scrutinize that foundation are discussed.

## Introduction

In their Moral Foundations Theory (MFT), Graham et al. (2013) proposed that people utilize the five foundations—(1) Care (not harming), (2) Fairness (not cheating), (3) Ingroup (not betraying), (4) Authority (not subverting), and (5) Purity (not contaminating)—to make moral judgments. The Care and Fairness foundations are referred to as the individualizing moral foundations, which focus on individual rights and autonomy, whereas the Ingroup, Authority, and Purity foundations are called the binding moral foundations, which focus on collective good and group coherence and hierarchy. The five foundations function as the standard for discriminating right from wrong in social situations. The ingroup members determine which of those five foundations are emphasized in organizing the group. People in a group often deal with morality-related situations by exchanging words about interpretations of those situations. In reinforcing the sharedness of the moral standard, language plays a key role in transmitting the set of moral norms shared in the group.

Language is crucial to human morality because it enables moral norms to regulate “should” and “should not” explicitly and the subsequent punishment system (Saucier, 2018; cf. DeScioli and Kurzban, 2018). Today, the linguistic communication of human beings is not limited to face-to-face physical interactions. Social media (e.g., blogs, Facebook, Twitter, and Instagram), in particular, has become a dominant mode of interaction in which physically distant people from various backgrounds can be connected. Cyber space may look different from physical space, but people express their experiences, feelings, policies, and values with language spontaneously in both spaces. Boyd and Pennebaker (2017) advocated that the digital world is where one’s personality would be embedded and that it is a useful platform to conduct personality research combining with self-report measures. For example, Qiu et al. (2012) investigated the relationship between personality and language use in microblogs and showed that personality traits, such as neuroticism, are expressed in linguistic patterns. As for morality, Brady et al. (2020) argued that social media is a place that enables moralized content to be addressed and transmitted because people tend to capture moralized content rapidly and spread moral-emotional content. It is necessary to be familiar with online language in relation to the social media platform that is being interacted with in order to know, understand, and interpret moral issues these days. People sometimes express their position about a moral issues and spread social issues by sharing it with others.

Research on people’s online language use in terms of morality has recently been accumulated with the help of Natural Language Processing and Text Mining (Kao and Poteet, 2007). Graham et al. (2009) developed a tool called the Moral Foundations Dictionary (MFD) to quantify the use of words in written texts referring to virtues and vices that are associated with each moral foundation. The MFD is a list of words that are relevant to each of the five foundations. There are virtue and vice word lists for each foundation. Combined with the Linguistic Inquiry and Word Count software (Pennebaker et al., 2001), the MFD enabled past empirical studies to quantify morality from written texts. For example, using this dictionary, Graham et al. (2009) analyzed the online text data of church sermons and found a higher frequency of words related to the Care, Fairness, and Ingroup foundations in sermons made by liberal preachers than those by their conservative counterparts (see also Takikawa and Sakamoto, 2019). Stolerman and Lagnado (2019) analyzed newspaper articles relevant to human rights and found that these articles contained more individualizing-related words (i.e., Care and Fairness). The previous research demonstrated that individual differences in political ideology are associated with language use involving morality. Later, Frimer (2020) replicated Graham et al.’s research and found the differences in language use focusing on political ideology are limited to religious contexts. Even the results that supported Graham et al.’s research showed small effect sizes. Frimer (2020) argues that liberals and conservatives may not speak different languages as much as researchers previously thought.

Although research on people’s language use between Democrats and Republicans has been accumulated, as mentioned above, the relationship between individual differences in terms of moral concern and the everyday online use of language has not been directly explored yet. It is important to cast light on individual differences as factors that affect morality-related language use because people’s political ideologies are associated with their moral concern (DeScioli et al., 2014). Understanding the patterns of the relationship between one’s moral concern as an individual difference and language use should help reveal how people interact publicly with others by using morality-related words in the digital space where they can choose to talk about anything. Individuals who personally rely on a certain moral foundation when making moral judgments can have a distinct pattern in their online language use. If this is the case, online language communication would be systematically framed by the communicator’s moral appraisal.

In addition, the effects of culture on the prioritized moral foundation(s) have been reported (Graham et al., 2011). The cultural effects are reasonable because moral judgment has an ultimate goal: to distinguish good from bad in a culturally correct way (Haidt, 2001; Haidt and Joseph, 2004). For example, people in the United States tend to interpret moral behavior from the view of the individual foundations, while Filipinos employ all of the foundations (Vauclair and Fischer, 2011; see also Vasquez et al., 2001). However, most morality research has been conducted in the so-called WEIRD (Western, Educated, Industrialized, Rich, and Democratic) cultural groups because many of the experimental measures, such as vignettes and video clips, are only available in English (Henrich et al., 2010; Clifford et al., 2015). The investigation of the use of language from the perspective of morality in a non-WEIRD culture is needed because it is not clear whether the findings obtained in research that was conducted in the WEIRD cultures can be generalized to other cultures.

Therefore, the present study is a preliminary investigation of the relationship between one’s moral concern as an individual difference and the use of language related to moral foundations in their everyday tweets among people in Japan. The newly developed Japanese version of the MFD (J-MFD; Matsuo et al., 2019) enabled the investigation. The present study employs a dataset that links the scores indicating one’s concern for the five moral foundations and their tweets: the first attempt to observe a specific moral concern guiding people to asymmetric language use on social media among Japanese.

## Methods

### Participants

Macromill, an Internet crowdsourcing service, recruited 386 Japanese participants who took part in our online survey. Among the original sample of 386 participants, 51 reported that they either did not have a Twitter account, had private accounts only, or refused to provide information about their accounts; 31 participants had accounts with zero tweets. The responses from the remaining 304 participants (386 − 51 − 31 = 304) were used for our data analysis (185 men and 119 women). Their ages ranged from 18 to 74 years old (*M* = 36.05, SD = 12.78).

### Materials

#### Moral Foundations Questionnaire

this questionnaire measures the degree of one’s concern for each of the five moral foundations based on MFT (Graham et al., 2011). To measure Moral Foundations Questionnaire (MFQ) scores in Japanese, we used the 30-item version of the Japanese MFQ (available at www.moralfoundations.org and Kanai, 2013). The Japanese version of the MFQ was found to have a five-factor model as the MFT predicted (Honda et al., 2016). Recently, Murayama and Miura (2019) determined the validity and reliability of the Japanese MFQ with large Japanese samples (855 Japanese participants in Study 1 and 470 in Study 2) and found that the five-factor model was the best fit. The Japanese MFQ has been used in previous research on morality conducted in Japan (e.g., Takamatsu and Takai, 2017; Matsuo et al., 2019). The Japanese MFQ consists of sentences that are rated on a Likert-type scale, ranging from 0 (not at all/almost never) to 5 (very much/almost always). There are six items for each of the five foundations. Responses are summed to create a foundation score for each foundation. A higher score indicates a higher level of concern for that foundation.

#### The Japanese Version of the MFD

Matsuo et al. (2019) translated the original MFD, which contains 324 English words, into Japanese using a semi-automated method. The authors provided evidence of its validity by comparing the number of the J-MFD words for each moral foundation in the situations that participants described as each foundation being followed and violated. The J-MFD includes 718 Japanese moral words with 11 categories corresponding to “Virtue” or “Vice” (violates); each is associated with one of the five moral foundations (i.e., Care, Fairness, Ingroup, Authority, and Purity) and included a more general or abstract category of morality (i.e., Morality General) (Matsuo et al., 2019). Because each English word in the original MFD possibly had more than one translated word in Japanese, the number of Japanese words in each category of the J-MFD became more than the original MFD. The J-MFD and a computer program for Japanese word segmentation are publicly available online^1^.

### Data Analysis

Looking at their Twitter accounts, we collected the tweets of the 304 participants (all the available user timelines) and applied the J-MFD to these tweets. The number of tweets per person ranged from 68 to 26,754 (*M*_tweets per person_ = 4,092.37, SD_tweets per person_ = 2,709.90). The number of word counts per person ranged from 2,440 to 1,202,062 (*M*_words per person_ = 145,565.62, SD_words per person_ = 133,017.30). We also collected self-reported responses to the MFQ from the same Japanese sample. We computed the “frequency ratio” of appearances of J-MFD words associated with each moral foundation (Virtue and Vice categories combined) as the same way Matsuo et al. (2019) did. We divided the word counts in Twitter by the size of the total words included in one’s tweets to obtain the ratio of J-MFD words in each participant’s tweet [e.g., the number of Care-related words in one’s tweets divided by the total word counts in his/her tweets, which is indicated as (i) here]. To obtain the ratio scores to be used for our data analysis, we divided that ratio of the J-MFD words [(i)] by the total number of dictionary words for each moral foundation [e.g., (i) divided by the number of the Care-related words in the J-MFD]. The relationship between the frequency ratio of J-MFD words and the MFQ scores was examined.

## Results

Tables 1, 2 show the means and standard deviations for the total rating scores on the MFQ by subscale and total word frequencies for each moral foundation. The correlations between the word frequencies for the Virtue and Vice categories for each moral foundation are shown in Supplementary Appendix A.

**TABLE 1:** MFQ correlations with means and standard deviations of its subscales.

**MFQ Subscale (*M*, SD)**	**Care**	**Fairness**	**Ingroup**	**Authority**
Care (24.47, 5.48)				
Fairness (22.61, 4.97)	0.77**			
Ingroup (20.03, 4.77)	0.45**	0.49**		
Authority (20.47, 4.70)	0.48**	0.56**	0.76**	
Purity (21.70, 4.61)	0.72**	0.69**	0.63**	0.66**

A higher score indicates a greater concern for the corresponding foundation. The score for each subscale ranges from 0 to 30. **p < 0.01.

**TABLE 2:** Correlations of word frequencies with means and standard deviations for each foundation.

**Moral foundation (*M*, SD)**	**Care**	**Fairness**	**Ingroup**	**Authority**
Care (0.00002, 0.00002)				
Fairness (0.00001, 0.00001)	0.61**			
Ingroup (0.00002, 0.00002)	0.65**	0.76**		
Authority (0.00002, 0.00001)	0.62**	0.77**	0.81**	
Purity (0.000008, 0.000005)	0.50**	0.42**	0.32**	0.28**

A higher score indicates a greater number of words for the corresponding foundation. The frequency ratio for each foundation ranges from 0 to 0.0002. **p < 0.01.

In Table 3, the five most frequent word in the total tweets by the J-MFD subscale is described.

**TABLE 3:** The five most frequent words in the total tweets by the J-MFD subscale.

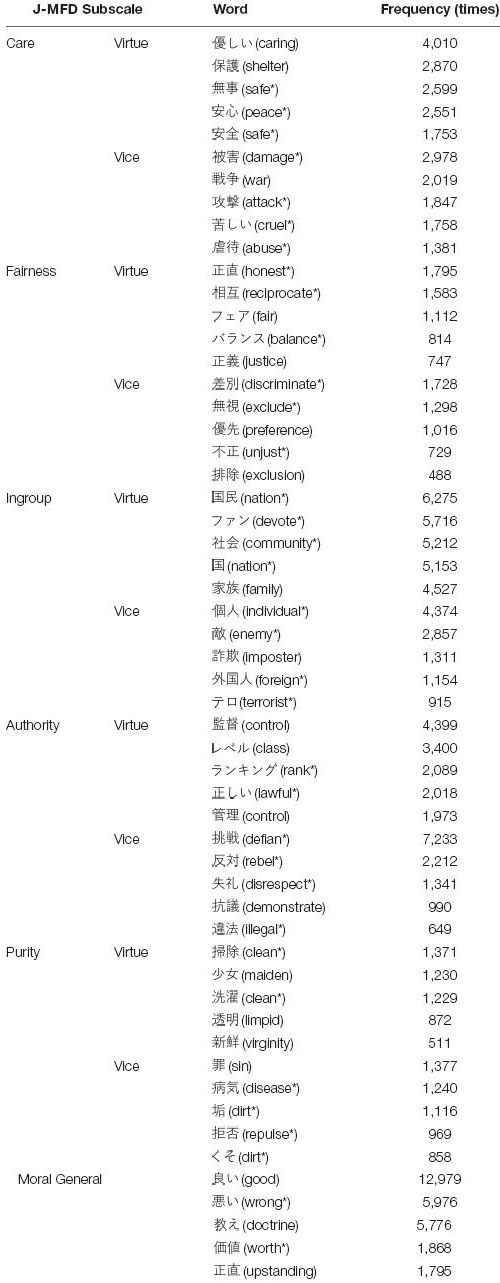

*Denotes a substring of more than one character.

A series of multiple regression analyses were performed to investigate how the frequency ratio of appearances of J-MFD words associated with each moral foundation was predicted by one’s moral concern represented by the MFQ scores. As shown in Table 4, there was a significant main effect for the MFQ Fairness score (positive effect) and for the Authority score (negative effect) on the word frequency score for all the foundations. Only for the Purity foundation were the main effects of the MFQ Care score (positive effect) and Purity score (negative effect) additionally observed.

**TABLE 4:** Summary of multiple regression and correlation analyses for variables predicting MFQ scores.

**Criterion variable**	**Predictor**	**β**	***t***	**95% CI**	***R*^2^**	***r***
Care-related Word Frequency	MFQ Care Score	0.08	0.81	[−0.11, 0.27]	0.08**	0.16**
	MFQ Fairness Score	0.31**	3.29	[0.12, 0.49]		0.20**
	MFQ Ingroup Score	−0.02	−0.18	[−0.19, 0.16]		−0.03
	MFQ Authority Score	−0.23*	−2.44	[−0.41, −0.04]		−0.06
	MFQ Purity Score	−0.05	−0.55	[−0.24, 0.14]		0.06
Fairness-related Word Frequency	MFQ Care Score	−0.10	−1.03	[−0.28, 0.09]	0.11**	0.13*
	MFQ Fairness Score	0.41**	4.48	[0.23, 0.59]		0.22**
	MFQ Ingroup Score	0.01	0.14	[−0.16, 0.18]		−0.03
	MFQ Authority Score	−0.34**	−3.71	[−0.51, −0.16]		−0.09
	MFQ Purity Score	0.09	0.94	[−0.10, 0.28]		0.09
Ingroup-related Word Frequency	MFQ Care Score	−0.03	−0.28	[−0.21, 0.16]	0.10**	0.18**
	MFQ Fairness Score	0.36**	3.86	[0.18, 0.54]		0.25**
	MFQ Ingroup Score	−0.002	−0.02	[−0.17, 0.17]		0.031
	MFQ Authority Score	−0.23*	−2.48	[−0.41, −0.05]		−0.00003
	MFQ Purity Score	0.07	0.68	[−0.12, 0.25]		0.14*
Authority-related Word Frequency	MFQ Care Score	−0.11	−1.15	[−0.30, 0.08]	0.10**	0.14*
	MFQ Fairness Score	0.40**	4.38	[0.22,0.59]		0.24**
	MFQ Ingroup Score	0.02	0.24	[−0.15, 19]		0.02
	MFQ Authority Score	−0.26**	−2.88	[−0.44,0.08]		−0.02
	MFQ Purity Score	0.08	0.80	[−0.11, 0.27]		0.12*
Purity-related Word Frequency	MFQ Care Score	0.21*	4.91	[0.02, 0.40]	0.09**	0.16**
	MFQ Fairness Score	0.23*	2.18	[0.04, 0.41]		0.16**
	MFQ Ingroup Score	0.08	2.43	[−0.09, 0.25]		−0.01
	MFQ Authority Score	−0.23*	0.90	[−0.41, −0.05]		−0.08
	MFQ Purity Score	−0.20*	−2.48	[−0.39, −0.01]		0.004

Coefficients are standardized; **p < 0.01 and *p < 0.05.

Thus, the results showed that the degree of concern for the Fairness and Authority foundations as the standards for moral judgment among Japanese individuals predicted their use of the words involving all of the five foundations.

A series of *post hoc* multiple regression analyses were conducted to investigate which Fairness-item(s) in the MFQ predicted the frequency of Fairness-related words in the J-MFD and which Authority-item(s) in the MFQ predicted the frequency of Authority-related words in the J-MFD. The results showed that the only Fairness-item in the MFQ (“When you decide whether something is right or wrong, to what extent is whether or not someone was denied his or her rights relevant to your thinking?”) significantly predicted the frequency of moral-related words across the five foundations on participants’ tweets (all *p* values < 0.001). Also, the only Authority-related item in the MFQ (“Respect for authority is something all children need to learn”) significantly predicted the frequency of moral-related words across four foundations on their tweets only Purity foundation was (non-significant, *p* = 0.07).

## Discussion

The present study aimed at exploring the effect of one’s emphasis on a moral foundation on their use of language on a social networking site among Japanese. The findings suggest distinct patterns in the interaction of language use and one’s concern for each moral foundation in the online communication setting. We found that one’s endorsement of the Fairness and Authority foundations predicted the frequency of words included in the J-MFD across all the five foundations.

In the present study, we found in our participants’ tweets that the most frequent Vice word for Fairness was “discrimination,” and that the words for Authority, Virtue, and Vice were “control” and “defiant”; these words, from those two foundations, are in conflict. Likewise, the Fairness item and the Authority item in the MFQ that best predicted the moral-relevant word frequency across the five foundations, are about having equal rights as others and the endorsement of authority from early childhood. Thus, the patterns of the results from the present study can be discussed from either of the perspectives of Fairness or Authority foundation because those two foundations are in a trade-off relationship. In fact, some previous research discusses these two foundations as the two sides of the same coin. The findings from Vecina and Piñuela (2017) suggested that endorsement of equal rights (i.e., Fairness foundation) is in conflict with someone else’s dominance (i.e., Authority foundation). Other previous research also discusses that it may be reasonable to regard these two foundations in the trade-off relationship rather than dealing with those foundations separately in isolation (Waytz et al., 2013; Monroe et al., 2020). Given the trade-off relationship, hereafter, we discuss our results from the Fairness perspective because it is generally easier to understand a positive relationship with moral-related online language use than a negative relationship between the concern for the Authority foundation and the use of language.

Our results show that Fairness concern is a strong predictor of moral-relevant word use in online language that occurs spontaneously. Indeed, Piazza et al. (2018) revealed that appraisals of injustice were, compared to the other kinds of appraisals (i.e., harm, disloyalty, authority, and impurity), the only significant predictor of intensity ratings of wrongdoing for all five moral foundations. Our findings may give further evidence to Piazza et al. (2018) by showing the universality of the centrality of Fairness in the MFT with another approach. Or it may be reasonable to suggest that the present results show Westernization among Japanese because Western people were found to be more appreciative of individual rights and justice (i.e., Care and Fairness foundations) than East-Asian people (Graham et al., 2013). Discussions suggest that the Japanese culture is becoming more individualistic than in the past (Matsumoto et al., 1996; Hamamura, 2012). Modern Japanese people may come to value equal rights and individual autonomy in the future. Because the present study recruited participants from all generations and all living areas, the trend of individualization (if any) should be attributed to Japanese society. The correlation between MFQ/MFD scores and age from our results (see Supplementary Appendix B) features the positive correlation between the MFQ Authority score and age; it may be a clue to capture possible differences in cherished moral values across generations in Japan. The present study cast light on the importance of Fairness, which can lead to future cross-cultural and longitudinal research to investigate this foundation.

Concerning the relationship between individual differences and behavior in language communication, Sylwester and Purver (2015) showed that conservative users of Twitter use more group-identity and religious words than liberal users, which reflects liberals’ tendency to emphasize the Ingroup, Authority, and Purity foundations less. As people’s interactions are widely expanded through cyberspace, it would not be surprising that their individual differences, such as moral concern, are disclosed online as shown in the present study that demonstrated that one’s language expressions are reflected by their tendency toward moral concern, which was not self-evident.

The present findings take initial steps toward a more comprehensive picture of how people communicate in moral terms and the effects of such communication on their actual behavior by incorporating traditional moral research ideas and methodology into the practical issue of the gap between inside and outside the lab. The Fairness Foundation is known as one of the moral foundations that are appreciated by liberals in the West (e.g., Graham et al., 2009). Henderson and Dressler (2019) state that the extent to which inequality can be endorsed is considered one of the most significant psychological dimensions that separate conservatives from liberals. Liberals are opposed to inequality, whereas conservatives have a higher tolerance for it because conservatives value authority more than liberals. On the other hand, differences in Japanese political ideology and the behavioral tendencies of the Japanese as a function of political affiliation are not transparent. Because Japanese people do not perceive themselves in a liberal vs. conservative framework, measuring their political ideology can depend on how researchers view Japanese political tendencies (Aoyama, 2019; Murayama and Miura, 2019). Future research will be enriched if the political ideology among the Japanese is captured in a deeper sense taking their unique culture into consideration.

People who are concerned with the Fairness or Authority foundation in Japan may be sensitive to the information about equality (cf. Gollwitzer and Bücklein, 2007). This tendency can be approached by the theory of justice sensitivity. Justice sensitivity is defined as a personality trait that puts importance on injustice and unfairness in everyday life (Schmitt et al., 1995). When this personality trait is connected with the notion of moral conviction, one’s motivational component concerning morality in the form of “oughts” or “shoulds” (Skitka and Mullen, 2002), people may declare what should be done to others. In today’s society, hate speech and SNS flaming exemplify the statement about one’s own “shoulds” for justice issues based on one’s moral conviction, which does not necessarily need reason or evidence. More recently, phenomena such as “refrain police” have been observed during the COVID-19 outbreak in Japan. Those “policemen/women” threaten others who violate the governmental self-restraint request based solely on their moral conviction. People who express their moral conviction specifically to show off their own moral quality by public discussion of morality and politics are referred to as moral grandstanders (Tosi and Warmke, 2016). These tendencies of moral conviction and moral grandstanding may hold their own set of moral norms about equality. It would be beneficial to investigate the mechanism and function of those tendencies within the framework of the MFT. On the other hand, Japanese people may have perceived the concept of fairness differently from Westerns in the first place (Kim and Leung, 2007). Thus, the Fairness foundation in Japan can be worth scrutinizing in future research.

Furthermore, the results showed a negative association between MFQ Authority and MFD Authority and the negative association between MFQ Purity and MFD Purity, which does not fit what one would intuitively expect. Given the zero-order correlations between the MFQ Authority and MFD foundation-related word frequency for all foundations and between the MFQ Purity score and MFD Purity-related word frequency, the MFQ Authority and Purity scores may serve as suppressor variables. If this is the case, at least it can be stated that the variables of the MFQ Authority/Purity scores can play an important role in predicting the MFD Authority/Purity-related word frequency (Knowlden, 2014). A better model with relevant variables will be constructed in future research.

A closer look at the Purity foundation can be another direction. We found a different pattern for Purity; only Purity-related online language use was predicted by one’s endorsement of the Care, Fairness, Authority, and Purity foundations. This finding may suggest that Purity has a distinct nature, compared to the other foundations, which has been explored in the previous research. For example, Kaur and Sasahara (2016) analyzed people’s tweets in English and found that Purity is a unique component separated by the other foundations. Also, Matsuo et al. (2017) discussed the possibility that Japanese people apply their unique lay theory to the interpretation of Shweder’s Divinity ethic (equivalent to Haidt’s Purity foundation). Their findings from the analyses of the participant-made moral transgressions suggest that Divinity violations and part of the Autonomy situations (equivalent to Haidt’s Care and Fairness foundations) are viewed under the same category in the Japanese cultural context. It may be hard to capture the possibly different nature of Purity among Japanese people with the Purity items on the MFQ because those items generally imply Christian concepts, such as the existence of only one God. Concerning this issue, Kitamura and Matsuo (2021) developed a tool to measure Japanese unique purity orientation, called the Purity Orientation-Pollution Avoidance Scale (POPA). The POPA scale measures Japanese people’s psychological pursuit for something pure and their avoidance of something polluted because of the experimental materials invented. This Japanese-customized scale would be a useful tool to quantify unique Japanese concepts of the pure and impure relevant to the Purity foundation in the MFT. Thus, the present study’s results may reflect difference(s) observed across cultures or a culturally unique Purity characteristic. The in-depth investigation of the perceptions of Purity among the Japanese should be awaited.

Overall, in the present study, online language use was found to reflect the users’ individual differences in moral concerns. The present study is a first step toward understanding how morality works among people in a non-Western culture. Using a promising tool such as the J-MFD, researchers interested in morality from any academic field can analyze texts that are filled with people’s mental representations about morality. In addition to controlled lab experiments, observations of people’s spontaneous expressions would be needed to clarify possible tendencies in language use because people do not randomly express their thoughts about morality. An investigation comparing Twitter users in English-speaking and Japanese-speaking cultures would also be beneficial. Any observed differences should be carefully scrutinized because those differences may come from using specific words with different connotations, not from the characteristics of cultures. Future research on morality—related to the sources of the differences—should investigate whether the present study’s findings can be extended with the modification or addition, or both of the original MFD and J-MFD. For example, Garten et al. (2016) attempt to extend the original MFD by combining the original MFD with the pre-trained distributed representations for words. Frimer et al. (unpublished data) developed a new MFD (MFD 2.0) by computing the prototypically of moral-related words that two of the authors selected. Garten et al. (2016) and Frimer et al. (unpublished data) based the selection of the words to be included in the MFD on the experts, which is similar to the development of the original MFD. On the other hand, Hopp et al. (2020) asked non-experts who were recruited through the crowdsourcing service to annotate moral-related words in news articles to extend the original MFD (eMFD). Our results in the present study would be analyzed more deeply (e.g., identifying prototypicality of each word in participants’ tweets) when the J-MFD takes those revisions of the original MFD into consideration. Because the J-MFD is open-access material and research can be revised, cooperating efforts to extend the J-MFD will enrich the analyses of text written in Japanese used for the research in various academic fields. For instance, like the extensions by the aforementioned studies, the J-MFD can be extended using automated and manual methods with the help of not only experts but also the layperson. Such extension will further contribute to identifying the mechanism and functions of human morality in their social life.

## Data Availability Statement

The materials, including tweet IDs and analysis programs, are available online at https://osf.io/nd59g/.

## Ethics Statement

Ethical review and approval were not required for the study on human participants in accordance with the local legislation and institutional requirements. The patients/participants provided their written informed consent to participate in this study.

## Author Contributions

KS devised the study and collected data. BD and AM analyzed the data. AM wrote the manuscript. KS helped with writing and editing the manuscript. All authors contributed to the article and approved the submitted version.

## Conflict of Interest

The authors declare that the research was conducted in the absence of any commercial or financial relationships that could be construed as a potential conflict of interest.

## Footnotes

1 https://github.com/soramame0518/j-mfd/
