# Supplementary material for: Appraisal of the Fairness Moral Foundation Predicts the Language Use Involving Moral Issues on Twitter Among Japanese
Source: Front Psychol. 2021 Apr 30;12:599024. doi: 10.3389/fpsyg.2021.599024 (PMC8120267; doi:10.3389/fpsyg.2021.599024)
Supplement: Supplementary file 1 [file Table_1.pdf]

Appendix A

Correlations between the word frequencies of the Virtue and Vice categories for the five foundations the J-MFD

Variable		Care Vice	Fairness Vice	Ingroup Vice	Authority Vice	Purity Vice
Word frequency	Care Virture	0.48**				
	Fairness Virtue		0.50**			
	Ingroup Virtue			0.64**		
	Authority Virtue				0.53**	
	Purity Virtue					0.28**

Note. ** indicates $p < .01$.

Appendix B

Correlations between the word frequencies and the MFQ scores for the five foundations and participants' age

Scale	Variable	Age
Word frequency	Age	
	Care	0.07
	Fairness	0.09
	Ingroup	0.11
	Authority	0.16**
	Purity	-0.16**
MFQ	Care	0.11
	Fairness	0.07
	Ingroup	0.02
	Authority	0.02
	Purity	0.16**

Note. **indicates p < .01.
